# Social Transmission and the Spread of Modern Contraception in Rural Ethiopia

**DOI:** 10.1371/journal.pone.0022515

**Published:** 2011-07-22

**Authors:** Alexandra Alvergne, Mhairi A. Gibson, Eshetu Gurmu, Ruth Mace

**Affiliations:** 1 Department of Anthropology, University College London (UCL), London, United Kingdom; 2 Department of Archaeology and Anthropology, University of Bristol, Bristol, United Kingdom; 3 Institute of Population Studies, College of Development Studies, Addis Ababa University, Addis Ababa, Ethiopia; Durham University, United Kingdom

## Abstract

Socio-economic development has proven to be insufficient to explain the time and pace of the human demographic transition. Shifts to low fertility norms have thus been thought to result from social diffusion, yet to date, micro-level studies are limited and are often unable to disentangle the effect of social transmission from that of extrinsic factors. We used data which included the first ever use of modern contraception among a population of over 900 women in four villages in rural Ethiopia, where contraceptive prevalence is still low (<20%). We investigated whether the time of adoption of modern contraception is predicted by (i) the proportion of ever-users/non ever-users within both women and their husbands' friendships networks and (ii) the geographic distance to contraceptive ever-users. Using a model comparison approach, we found that individual socio-demographic characteristics (e.g. parity, education) and a religious norm are the most likely explanatory factors of temporal and spatial patterns of contraceptive uptake, while the role of person-to-person contact through either friendship or spatial networks remains marginal. Our study has broad implications for understanding the processes that initiate transitions to low fertility and the uptake of birth control technologies in the developing world.

## Introduction

The decrease in fertility rates accompanying the increase in wealth and well-being of societies, i.e. the demographic transition, has attracted a great deal of interest among scholars from both social and biological sciences [1], [2], [3]. While diverging in their approach, a common interest of those fields is to understand the role of individual vs. cultural factors in explaining reproductive decisions. Understanding how behaviours that appear a priori to be maladaptive at the individual level (i.e. reducing reproductive success [4], but see [5]) presents a particular challenge to evolutionary anthropologists, some of whom have argued that an increase in frequency of maladaptive behaviours can arise as a response to social transmission, i.e. the diffusion of ideas and behaviour through social interactions [6], [7].

It is widely agreed that fertility decline is not simply an adjustment to changing socio-economic circumstances, and that additional understanding can be gained by taking into account the social transmission of fertility ideas and behaviours [8], [9]. In particular, social diffusion has been invoked to explain why, among both preindustrial and industrial countries, fertility varies widely at any given level of development [10]. Social transmission has been suggested to explain the spread of low fertility norms during the European demographic transition: using provincial data (1870-1960), Coale & Watkins [11] showed that, once a region in a country has began to decline, neighbouring regions with the same language or culture follow after short delays, even if they were less developed. It has thus been argued that fertility decline reflects the spread of key attitudes (e.g. about the ideal family size) and behaviours (e.g. uptake of birth control technologies), a process partly independent from societal structural changes (e.g. decrease in mortality rate, availability of contraception), which can account for a unique portion of the variation in the timing and pace of change [10].

Diffusion refers to the process by which innovation spread among regions, social groups or individuals [12], and in particular, “diffusion exists when the adoption of innovative ideas (and corresponding behaviour) by some individuals influences the likelihood of such adoption by others” [13]. Individuals are embedded in a network of social relations, and social interactions can influence both access to information as well as the intensity of control exerted to enforce social norms. In particular, social interactions may provide a venue for payoff biased social learning and/or social influence (e.g. conformism; [10], [14], [15]). While *social learning* emphasizes the role of information in reducing uncertainty associated with the innovation [16], *social influence* refers to the process through which some individuals exert control over others, by virtue of their power or authority (although social influence may be hard to distinguish from prestige biased social learning [17]). Social effects (i.e. social learning and social influence) can accelerate or retard the process of fertility change. For instance, social influence is likely to be a critical factor in maintaining high fertility at early stage of fertility transition [18], and as such has been a topic of great interest to demographers.

There is accumulating evidence that the deliberate control of fertility within couples may be an innovation that diffuses through social interactions [13], [14], [19], [20], [21], [22], [23], [24]. However, most previous studies are cross-sectional, thus precluding ruling out the possibility that social interactions and fertility are jointly determined: unobserved factors might affect both behaviour and choice of social network partners [25]. For instance, as members of a social network are usually spatially aggregated, increase in the prevalence of contraceptive uptake in both individuals and their networks might result from extrinsic factors associated with local conditions (e.g. access). Additionally, women wanting to use modern contraception might be more inclined to choose networks partners who use family planning and indeed several studies specifically asked women to define their networks in terms of whom they talked to about contraception. Such family planning networks contain a high prevalence of individuals who can provide information (i.e. contraceptives users [26], [27]). Therefore, any effect of social transmission is more likely to indicate information seeking, resulting from an individual decision to adopt contraception, rather than the imitation of others' choices. Because family planning networks underestimate the occurrence of social diffusion through copying relatively to information seeking, the possibility that fertility restriction can spread as a result of imitation cannot be adequately investigated. Rather, considering social transmission from and to networks of individuals with whom one normally interacts might prove more relevant, as those networks are not biased towards women *a priori* contemplating the adoption of modern contraception.

Whilst macro-level studies have revealed evidence for social diffusion of contraceptive technologies, as well as for socio-economic determinants (like education and low mortality; e.g. [11], [13]), there has been surprisingly few micro-level studies on how this particular innovation spreads through communities at the local level, and even fewer have caught the first cases of the new innovation, when the nature of the spread should be most easily determined. This study investigates the extent to which considering social interactions improves our understanding of temporal and spatial patterns of first contraceptive uptake, using data from rural Ethiopia. The situation of Sub-Saharan countries is of particularly interest as their low prevalence of modern contraception is thought to result from social influence [8]. In Ethiopia for instance, contraceptive uptake is still low (15% and 10.9% in urban and rural areas, respectively; [28]) despite the government having a specific population policy to promote voluntary contraceptive since 1993.

The objectives of the study, covering a period of 14 years, 4 villages and involving >900 women, are threefold: (i) to describe the temporal and spatial patterns of uptake of modern contraception, (ii) to identify the individual characteristics that predict the adoption of modern contraception (iii) to investigate whether and to what extent uptake of modern contraception is predicted by social effects (e.g. imitation and/or information seeking) once individual characteristics are taken into account. Our data have at least three strengths to understand the role of social diffusion for fertility decisions. First, network data contain information on both spatial relationships and friendships networks. In particular, women and their husbands were asked to name up to 5 other same-sex individuals with whom they talked most and perceived as their best friends (be they related, unrelated or living near or far away). Second, for each woman, the year of first use of contraceptives is informed along with network data. This allows to consider the timing of adoption events within a given network, and thus to infer the sequence of events. Finally, the study covers a period including when contraceptive use is just beginning, thus providing information on the role of social transmission at an early stage of the diffusion process when patterns of diffusion should be clearest.

## Materials and Methods

### 1. Study site

The study is based on a community of agro-pastoralists living in 4 villages of the Arsi Administrative zone, southern Ethiopia. In this rural area, the resources are limited, and the community suffers from periodic shortages of both water and food. Access to basic health service and school is restricted: the nearest health care services and high schools are over 20 km distance from the villages (see [29], [30] for more information in the study site). Interestingly, although contraceptive prevalence is generally low among rural women (i.e. <3% in 2003), there is now evidence that contraceptive technology is gaining in popularity and demand for family planning is increasing. Informal focus groups on contraception conducted in 2005 conducted by RM and EG have revealed that the level of interest expressed in contraceptive use was far greater than anticipated given the low prevalence in 2003.

### 2. Data Collection

A total of 943 ever married women of reproductive age (15-52) residing in the four villages were interviewed in 2008/9 about their birth and contraceptive histories. This includes all women living in the villages at the time of the survey as identified by a census of the four villages in 2008. The survey included questions on whether women had ever heard about modern contraception, and whether they had ever use it in the past (even though they might not be currently using it) and when (i.e. before their first birth, or after which birth). This allowed to identify the first year of contraceptive uptake and to reduce the uncertainty generally resulting from recall data. For those women who had ever used contraception, method and place of delivery were also informed. The quality of the data has been checked through comparisons with previous surveys conducted since 1999, date at which the process of contraceptive uptake was at an early stage. Only women for whom age and birth history record was known were included in the analysis. This reduced the sample to 936 women (among which >99% were married by 2008). For each woman, information on her demographic and socio-economic characteristics, her friendship social network, her husband's friendship social network, and her spatial location were collected (see next sections). Clearance was obtained from the UCL Research Ethics Committee and the Ethiopian Science and Technology Agency (ESTA). Signed consent was obtained from all participants. Those unable to read were read the forms before signing. The procedures were approved by the UCL and ESTA ethics committees.

#### (a) Demographic and socio-economic variables

Each woman was asked about her age, marital and birth histories, education (binary variable: attended formal school or not), social status (2 variables to take into account variability in the extent to which individuals rely on crops or pastoralism: the first calculated based on the amount of crops in kg harvested the year before (teff, wheat, maize and barley), weighted by price given by the Food and Agriculture Organization of the United Nations (http://www.fao.org/), the second based on men's cattle possessions), religion (i.e. Muslim or Orthodox Christian), ethnic origin (i.e. Arsi or Shoa Oromo), and her use of a radio (i.e. never, sometimes, frequently). Women were assigned to different cohort depending on their year of birth (4 levels: ≤ 1960; ≤ 1967 & >1960; ≤1973 & >1967; >1973).

#### (b) Friendships networks

To record data on friendship networks, women were asked the following question “Name up to 5 other women with whom you talk most and perceive as among your best friends”. Similarly, men (*N* = 869, 79% of husbands found) were asked to name up to 5 such men. This allows us to draw a matrix of whom-to-whom communication, the elements of the matrix weighting the strength of the ties (1 if a contact was formed, whether or not it is reciprocated, or zero otherwise). It could be argued that social influence is stronger among reciprocated ties, yet studies on very large datasets did not find any significant differences in social influence between reciprocated and non-reciprocated ties [31], [32]. Information was also collected on the relationship to each friend/relative named, and whether these friends lived in or outside the study area (See Table 1). Among women's friends, ∼9% are kin and ∼52% are affines, while among men's friends, ∼21% are kin and ∼41% are affines. Of those named, 19.9% of men's networks and 12.2% of women's networks were outside the study area and could not therefore be identified, leading to a “quasi-complete” social network. In total, 8.7% of women do not have a recorded social network and 31.7% do not have a network recorded for their husbands. Social networks provide information on both the structural properties of individuals (i.e. centrality or number of connection), as well as the content properties of their networks regarding reproductive behaviour (i.e. the proportion of ever-users) at time *t-1*. Since it is unlikely that adopting modern contraception at time *t* influence the proportion of ever-users in a network at time *t*-1, any effect is more likely to be interpreted as an influence of the network on the focal individual rather than the reverse.

**Table 1:** Characteristics of friendship networks for women (female friends) and their husbands (male friends).

	Women	Men
**Mean no. of friends (range)**	2.68 (0–5)	3.82 (0–5)
**Relationship (%)**		
Mother/Father	01.85	01.72
Sister/Brother	07.10	19.35
Co-wife	05.82	---
Spouse's mother/father	16.24	01.74
Spouse's brother's wife/sister's husbands	29.78	01.90
Spouse's sisters/brothers	06.53	37.10
Friend	09.00	23.86
Neighbour	18.13	06.08
Not stated	05.54	08.25
**Location (%)**		
Same compound	50.31	07.04
Same village	43.59	87.37
Other village	06.10	05.59
**Contact Frequency (%)**		
Everyday	76.25	72.66
Once a week	17.70	12.32
Once a month	02.79	02.03
Less than monthly	03.26	12.99

Women are more likely to appoint affines as their friends (e.g. their husband's brother's wives (29.78%) and their husband's mother (16.24%)). Husbands appoint affines (spouse's brothers (37.10%)), friends (23.86%) and kin (e.g. their brothers (19.30%)). Women's friends are more likely to be in the same compound, while men's friends are more likely to be in the same village but outside the compound. In more than 70% of cases, women and men talk to their friends every day.

*Structural properties*. Two variables were used to describe the structural position of individuals within their networks. First, the number of nominations received (i.e. indegree) informed on how integrated an individual is in a network, and is usually referred to as a measure of opinion leadership or popularity. It is often argued that individuals who are highly interconnected are more likely to hear about innovations earlier and to have more opportunities for social comparisons and influence [12]. Second, the number of nominations sent (i.e. outdegree) allows to control for the size of the network, and thus for any unobserved characteristics that lead to differential efforts by women in building and maintaining social interactions. Each woman's in-degree and out-degree were derived at the community level (all villages included).

*Content properties*. For each woman and at each time step, a variable describing her exposure to modern contraceptives through either her network or, if married, her husband's network (through wives of her husband's friends) was built. Note that at any given time, women were eligible to be part of a network only if they were aged at least 15. Specifically, at any given time *t*, the proportion of ever-users in a woman's network corresponds to the proportion of individuals in the network that had ever used contraceptives at time *t*-1.

We created variables to inform on both unbiased and biased social influence. First, all network members were assumed to have the same influence (i.e. unbiased transmission or conformism, all ties between friends equal 1). Second, to take into account that ever-users in a network can have different influence depending on their prestige ([17]; biased transmission based on popularity, social status and/or education), the ties linking two individuals have been weighted according to standardized coefficients describing the prestige of individuals (varying between 0 and 1, value divided by the maximum value in the population).

#### (c) Spatial networks

A matrix was built that describe the spatial distance between each pair of points. Spatial coordinates associated with each household where individuals lived were recorded using a Global Positioning System (GPS type Garmin) using longitude and latitude coordinates (using degrees minutes and seconds (DMS) nomenclature). DMS were converted to Decimal degree to calculate distance between each pair of points using the great circle distance calculation (exact distance is calculated using spherical trigonometry, http://www.zipcodeworld.com/docs/distance.pdf). For each woman at each time *t*, the minimum distance to an ever-user at time *t-1* was inferred.

### 3. Statistical analysis

#### (a) Model

Since the data are right-censored (some women were non-ever-users at the last time period), contain a large amount of ties (many women had the same adoption time), and are recorded on an approximate time scale (years), data were converted into a person-period life table on which a discrete time hazard model was performed (i.e. a logistic regression including the main effect of period [33]). For each woman, the beginning of time (Period 1) corresponds to the first year of eligibility for the use of contraceptive: either the year of the first adoption event ever recorded (1995) if aged at least 15 at that time (75% of women), or, for younger women, the year at which they reach the age of 15. Once they have adopted modern contraceptives, individuals exit the dataset. The population value of discrete-time hazard for woman *i* in time period *t* is thus the probability that she will experience contraceptive uptake in that time period, conditional on no prior event occurrence and her particular values for the predictors in that time period. Variables included are both time-invariant (e.g. use of radio, village) and time varying (e.g. age, parity and proportion of ever-users in networks). The proportionality assumption (that the hazard risk of contraceptive uptake is independent of time for any given variable) has been checked for key variables (e.g. parity, proportion of ever-users in the social network).

#### (b) Model Selection

A set of a priori candidate models is assumed (see next section), for which a measure of each model's fit scaling to its complexity is derived (e.g. Akaike information criterion [34]). The model for which AIC is minimized is selected as the best for the empirical data at hand. The evidence for each alternative model is done by rescaling AIC values relative to the model with the minimum AIC, which subsequently allows models to be ranked according to their ability to account for the data. In addition, a measure of weight of the evidence that a given model is the best in the set of models considered is calculated (Akaike weight (ω)). Subsequently, rather than base inferences on a single selected best model, inferences are calculated using the entire set using model-averaged based estimators (estimates are balanced using Akaike weights and averaged across models; [35]). The use of model based average estimators allow better precision and reduced bias compared to the estimator of that parameter only for the best selected model. Following [36], we present only models that collectively account for 95% of the available model weight. All analyses were carried out using R software (version 2.11.0).

#### (c) Candidate models

*Individual characteristics (set 1).* If women plan the size/composition of their family so that they maximize their number of surviving offspring and/or surviving males, one can expect the adoption of modern contraception to be positively associated with the number of living children (Parity) and/or the proportion of living sons (Prop_LS), as well as negatively associated with the number of deceased offspring (Nb_DO). These possibilities are investigated with models controlling for the quadratic relationship between a woman's reproductive history and her age, her cohort, as well as for her marital status (MS):

(i) *Age + Age^2^ + MS + Cohort + Parity*,

(ii) *Age + Age^2^ + MS + Cohort + Nb_DO*,

(iii) *Age + Age^2^ + MS + Cohort + Prop_LS*,

(iv) *Age + Age^2^ + MS + Cohort + Prop_LS + Nb_DO*,

(v) *Age + Age^2^ + MS + Cohort + Parity + Nb_DO*.

Additionally, contraceptive uptake is generally found to be associated with a woman's level of education [37], with educated women more likely to delay or reduce their reproduction in order to pursue their studies, or more informed on contraceptives. The variable describing women's level of education (Edu) is thus included to all 5 previous models.

(vi) *Age + Age^2^ +MS + Cohort + Parity+ Edu*,

(vii) *Age + Age^2^ + MS + Cohort + Nb_DO + Edu*,

(viii) *Age + Age^2^ + MS + Cohort + Prop_LS + Edu*,

(ix) *Age + Age^2^ + MS + Cohort + Prop_LS + Nb_DO + Edu*,

(x) *Age + Age^2^ + MS + Cohort + Parity + Nb_DO + Edu*.

Finally, we investigated the relationship between wealth and the adoption of contraception uptake. A first scenario is that women from poor families experience a stronger trade-off between quantity and survival of offspring, and are thus expected to adopt modern contraception earlier. This has been found to be the case in rural Gambia [38]. Alternatively, in populations experiencing a decrease in child mortality as a result of modernization, it has been argued that the trade-off between fertility and parental investment increases with family socio-economic status. We investigated the relationship between socio-economic resources and contraceptive uptake in including material wealth variables (husband's cattle, and agricultural production) to all previous models (note that the set also contains a null model).

(xi) *Age + Age^2^ + MS + Cohort + Parity + Edu + Wealth*,

(xii) *Age + Age^2^ + MS + Cohort + Nb_DO + Edu + Wealth*,

(xiii) *Age + Age^2^ + MS + Cohort + Prop_LS + Edu + Wealth*,

(xiv) *Age + Age^2^ + MS + Cohort + Prop_LS + Nb_DO + Edu + Wealth*,

(xv) *Age + Age^2^ + MS + Cohort + Parity + Nb_DO + Edu + Wealth*.

*Individual characteristics, social environment and social interactions (set 2)*. Individuals' decision to adopt modern contraception might be better understood if one considers not only socio-demographic determinants of fertility but also individual's social environment (e.g. group norms and media exposure). For instance, contraceptive uptake possibly differs among religious groups, as it has been shown to be the case in many countries (in Europe [11], in Ghana [39], in Malawi [37]). Note that variation in religious norms is observed in only 1 village, with all other 3 villages being characterized by a 100% Muslim obedience (See Table S1). Also, access to mass media and programs/adverts on modern contraception through the use of radio might exert a social pressure on women and/or provide them with more information hence reducing risk perception. In both cases, access to mass media is predicted to positively influence uptake of modern contraception. Models including social environment factors (SE) and/or individual factors (IF) were thus considered: (*i) IF; (ii) SE; (iii) IF + SE,* with *SE* referring to *Religion + Radio + Village.* Individual's decision to adopt modern contraception has also been suggested to partly result from social diffusion, i.e. to be related to social interactions (SI) or person to person contact, which lead to the inclusion of the following models in the set (*iv) SI; (v) SE + SI; (vi) IF+ SI; (vii) IF + SE + SI.*

Several variables were considered to describe social interactions (SI), including spatial distance to contraceptive ever-users and both structural and content properties of friendships' networks. First, we investigated whether women are more likely to adopt contraception at time *t* if they are spatially close to individuals having already used contraception at time *t-1*. This was done by considering the minimum spatial distance to a contraceptive ever-user (SI1: Min_Distance). Second, we investigated the role of women's centrality, i.e. the number of nominations received through both women's and husband's network (SI2: Indegree_W_NT_, Indegree_H_NT_). Indeed, central individuals are more likely to hear about innovations [40] and are in turn likely to adopt contraception earlier. Third, we investigated the role of the content of the friendship network. We considered the proportion of ever-users in women's and/or their husbands' social network at time *t-1*, with both unbiased and biased transmission according to characteristics of network partners (SI3: unbiased transmission: Prop. ever-users_W_NT_ + Prop. ever-users_H_NT_; SI4: biased transmission according to popularity; SI5: according to education; SI6: according to wealth). Indeed, some friends might be more influential than others [17]. Fourth, models assuming an interaction effect between social interactions and individual factors were also considered. Indeed, the role of social networks might depend on the “readiness” of individuals to adopt an innovation. For instance, women willing to adopt modern contraception as a result of individual circumstances might be more likely to do so if they can get information or receive positive attitude from other network members. Those models include an interaction effect between the number of living children (Parity) and the proportion of ever-users among network members at time *t-1*. A total of 32 models were included in the set.

## Results

**Figure 1:**
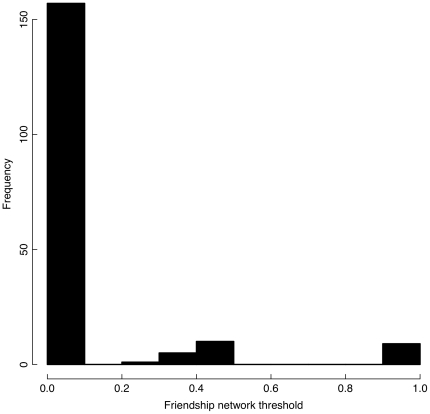
Thresholds for contraceptive uptake in women's networks at the time of adoption. A threshold corresponds to the proportion of adopters in an individual's network at the time of adoption of an innovation. Among women who have ever used contraception (*N* = 176), 86.3% are innovative relative to their network members, while 89.0% are innovative relative to their husband's networks.

The risk of contraceptive uptake is best predicted by a combination of socio-demographic (i.e. the number of living offspring and education level) and social environment (i.e. religion and media exposure) characteristics. Social relationships with best friends and proximate households, however, do not improve the understanding of temporal and spatial patterns of contraceptive uptake. Interestingly, at the time of adoption, most women (>85%) are innovators relative to their friendship networks (e.g. all network partners have never used contraception in both women's and their husband's friendship networks, Fig. 1), and there is no evidence of a negative influence of non-adopters on contraceptive uptake. Rather, if relevant at all, the role of social interaction through friendships networks appears to be conditional of individual circumstances, some individuals being more sensitive to social transmission than others (i.e. women with the higher number of children). The study tells about a population in the early stages of contraceptive uptake when patterns of social diffusion are most tractable; results in populations where contraception is already widely used may differ.

### 1. Contraceptive uptake: temporal and spatial patterns

**Figure 2:**
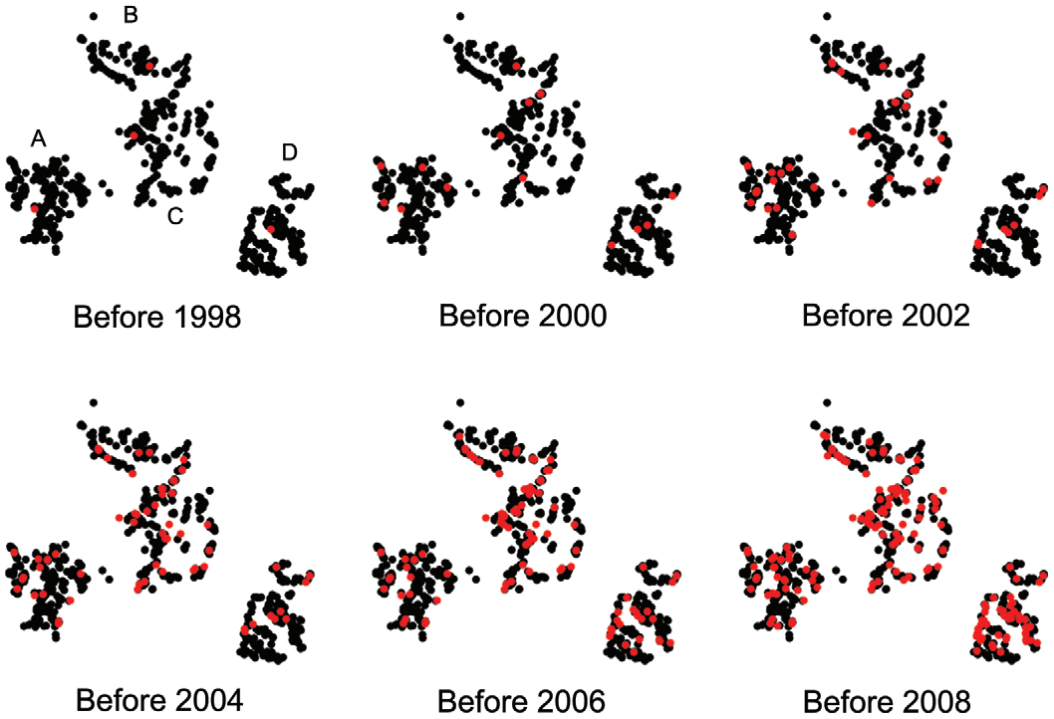
Temporal and spatial patterns of contraceptive uptake. Contraceptive prevalence varies from less than 1% before 1998 to reach a level of 18.8% in 2008 among women of reproductive age (i.e. 15-45 years, *N* = 936). Adoption of modern contraception also shows spatial variation (i.e. village A = 22.3%, village B = 15.4%, village C = 22.5%, village D = 22.8%).

The earliest evidence of contraceptive uptake occurred in 1995. Contraceptive uptake increased and reached the level of 18.8% by 2008 among women of reproductive age (15-45 years). At that time, 96% of women had already heard about contraception, although it does not necessarily translate into behavioural change. The number of women having ever used contraception varies from 15.4% to 22.8% across the 4 villages considered (Table S1, Fig. 2), and the most frequent methods used are pills (30%) and injection (70%). Women generally use contraceptives for the first time after they have already reproduced (in 95.6% of cases). The mean number of children (± s.d.) at the time of adoption is 3.7±2.1, which is consistent with the idea that women use contraceptives to either space births and or to end their reproductive career once they have achieved their desired family size. Note however, that 4.4% of the total number of ever-users reported having used contraception before their first birth.

### 2. Decisions for contraceptive uptake

Using data covering a period of 14 years (1995-2008), we investigated a blended model of fertility dynamics (e.g. an extended version of Cleland's model [8]), in which both individual and social factors matter. We used information theoretic methods (i.e. model comparison), providing a strength of evidence for an a priori set of alternative hypotheses [35]. A first set of models was considered to identify the most likely individual characteristics to account for patterns of contraceptive uptake. Second, these individual factors were combined with social environment variables (e.g. religion) and/or social interactions (e.g. centrality measures, proportion of ever-users among friendship networks at time *t-1*, minimum spatial distance to an ever-user at time *t-1*) in a second set (see Methods). This procedure limits the number of models considered, and thereby increases the probability that model selection reflects the genuine contribution of variables rather than spurious effects [35].

#### (a) Individual characteristics

**Figure 3:**
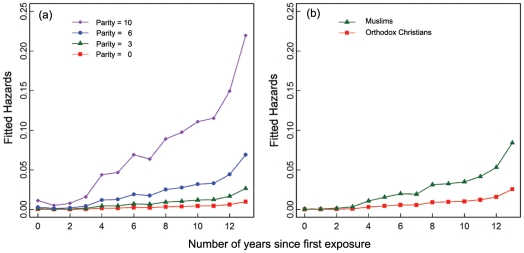
Risk of contraceptive uptake across time: main predictors. (a) Parity (number of living children). (b) Religious group. The risk of contraceptive uptake increases by 40% with each additional child. As compared to Muslims, Orthodox Christians show a 80% decrease in the risk of contraceptive uptake. The relationships are controlled for age, age^2^, social status, cohort, education and marital status.

Model ranking reveals that the best model to account for contraceptive uptake (Akaike weight  = 0.49, Table S2) includes the number of living children (parity: OR  = 1.43; 95%CI [1.28; 1.62], Fig. 3a), the level of education (OR  = 2.16; 95%CI [1.53; 3.03]), while controlling for age, age^2^ and marital status (polygynous as compared to monogamous: OR  = 1.22; 95%CI [0.85; 1.77]) and cohort effects (OR  = 2.80; 95%CI [1.82; 4.34]). The number of children deceased and the amount of material wealth have no influence on contraceptive uptake while the number of living sons has a marginal positive effect (OR  = 1.54; 95%CI [0.99; 2.41]), suggesting that sons are preferred over girls (Table S3). That material wealth is independent form contraceptive uptake is intriguing, as it has been shown to have effects, either positive [41] or negative [38] in other studies.

#### (b) Additional role of social environment and social interactions

**Figure 4:**
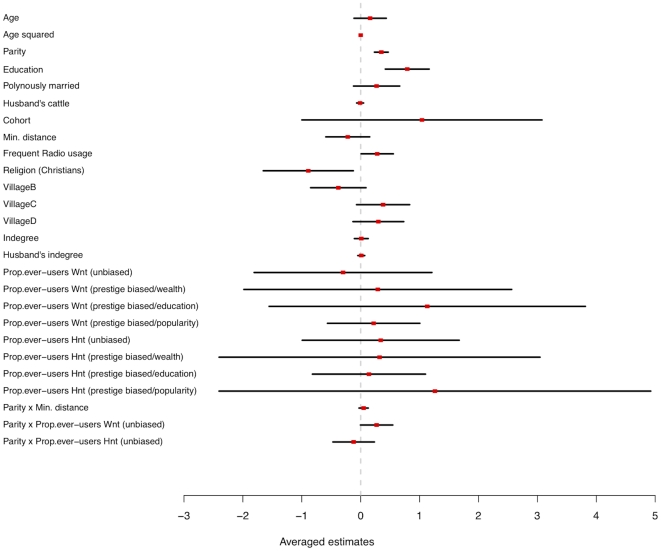
Averaged estimates (red squares) and 95% confidence intervals (black lines) for the effects of individual factors, social environment, and social interactions on the risk of first contraceptive use. Data cover a period of 14 years and involve >900 women (see Methods). Wnt: women's network; Hnt: husband's network; formal education is compared to the level “no education”; villages are compared to the first level “Village A”; Being polygynously married is compared to being “monogamously married”. “x” indicates an interaction.

First, model ranking shows that individual factors and social environment should both be considered, as this combination provides the best account for the data at hand (sum of Akaike weight of models including these variables >0.95, Table 2). Furthermore, model comparison shows that models excluding either group of variables account for less than <1% of the total weight of the set. Including religious and village characteristics to a conventional socio-demographic model improves the understanding of the data. While controlling for individual factors, averaged estimates show that Orthodox Christians are less likely to adopt modern contraception than Muslims (OR  = 0.22; 95%CI [0.10; 0.52], Fig. 3b, Fig. 4). Interestingly, shorter birth intervals have been reported for Orthodox Christians [30] in this population, indicating a higher emphasis on fertility in this religious group. Finally, women frequently listening to the radio are more likely to be ever-users (OR  = 2.12; 95%CI [1.18; 3.78], Fig. 4), independently of individual wealth differences as they are controlled for. Note that radio use only informs on individual's behaviour in the year of interview, which precludes to conlude on any causality effect.

**Table 2:** Best models for contraceptive uptake.

Models	K	LogLik	dAIC	ω_i_
IF + SE	28	-790.34	0.00	0.29
IF + SE + SI (Min. distance)	28	-789.49	0.28	0.25
IF + SE + SI (Min. distance) × Parity	30	-788.72	0.74	0.20
IF + SE + SI (Biased prop. ever-users (popularity))	30	-789.92	3.16	0.06
IF + SE + SI (Biased prop. ever-users (wealth))	30	-790.25	3.81	0.04
IF + SE + SI (Unbiased prop. ever-users)	30	-790.27	3.86	0.04
IF + SE + SI (Biased prop. ever-users (education))	30	-790.30	3.92	0.04
IF + SE + SI (Centrality)	30	-790.34	3.99	0.04

K: number of parameters; LogLik: Loglikelihood; dAIC: deviation from the best model's AIC; ωi: Akaike weights; IF: individual factors; SE: social environment; SI: social interactions, “x” indicates an interaction term. See Fig. 4 for averaged estimates and confidence intervals of variables, and Methods for more details on the candidate models.

Second, social interactions through friendships networks and geographical proximity do not improve the understanding of the timing of contraceptive uptake once individual factors and social environment are taken into account (Table 2). It could be argued that information on friendship network is biased, as composition might have changed across years or with migration. This possibility has been checked conducting an analysis restricted to the year of the interview and similar results have been obtained (Table S4). If the structure and the content of networks are not found to be critical, they are informative (Fig. 4). Interestingly, social effects appear to be conditional of both the type of network and individual characteristics.: women's social network matter more for those having a high number of living children (Parity × unbiased Prop. ever-users Wnt: OR = 1.30; 95%CI [1.14; 1.51]). Overall, the results support the idea that person-to-person effects on contraceptive uptake reflect a social learning process (rather than social influence), although the magnitude of the effect is weak and there is a high uncertainty among models including social interactions (Table 2). Finally, there is no effect of the number of individuals listing you as a friend (Indegree), so popular individuals, who may be among the more prestigious in the population, are not more likely to be ever-users than anyone else. Moreover, they are not more likely to influence others (no effect of prestige-biased friendship networks; Fig. 4).

## Discussion

Social diffusion of fertility values and behaviours has been invoked to account for macro-level patterns of “contagion” observed during the demographic transition [11]. More generally, it has been suggested that social transmission through social interactions explains why cultural traits that do not enhance individual reproductive success may spread [6]. In this paper, we used data from a population entering the demographic transition to understand the role of social transmission for the early spread of modern contraception, a cultural variant a priori maladaptive (but see [5]). In particular, we investigated the extent to which a woman's decision to adopt modern contraception is the result of (i) the proportion of ever-users/non ever-users within both women and their husbands' friendships networks and (ii) the geographic distance to contraceptive ever-users. We found that decision to adopt modern contraception is strongly determined by individual demographic and socio-economic characteristics, and by the religious group. However, we show that the contribution of social transmission from either best friends or contraceptive ever-users in proximate households is minimal. Whether other sources of social transmission (e.g. from doctors [18], husbands [42], religious leaders [43], kin [24], [44], [45] or weak ties [46]) could affect the pace at which women adopt modern contraception is discussed. The study can only tell us about a population at the early stages of the demographic transition, and mechanisms at later stages or in populations characterized by higher economic development might differ. Yet, the results are important if one is to understand why women adopt modern contraception in the first place.

The results show that the early rise in modern contraception is mainly predicted by individual demographic and socio-economic characteristics. First, contraceptive uptake increases with family size, and to a lower extent, with the number of surviving males. One plausible explanation is that contraceptive uptake is driven by competition for resources and need for higher level of investment per child as the society enters the market economy [47], [48]. Interestingly, sibling competition for education, particularly between brothers, is argued to have recently increased in the population as a result of modernization [30] and in response to land shortages [49]. Women might also perceive higher rates of mortality with increasing parity, which is not inconsistent with a quantity/quality trade-off perspective since maternal mortality is a major cause of death in young children [50]. At a proximate level, women might grow tired of giving birth but also experience higher social status and autonomy with increasing parity, thereby facilitating contraceptive uptake. Alternatively, the link between parity and contraceptive uptake could result from social transmission, e.g. if high parity women interact more frequently with medical centres in which antenatal care is delivered. Whether this is the case, and whether such social transmission reflect individual social learning (i.e. information seeking) and/or social influence remains to be tested. Second, the results show that, as in modernized societies [51], educated women are more likely to start using contraception: among ever-users, 71% are educated. Note that no women in this population continue to study after marriage, so educated women do not adopt contraceptives to pursue education. Rather, the higher prevalence of contraceptive use among educated women might reflect higher knowledge about contraceptives and/or social influence of teachers. Overall, the results suggest that birth control decisions are shaped by individual variation in both reproductive trade-offs and opportunities for social transmission.

We found evidence that the early spread of modern contraception is best understood if both individual socio-demographic characteristics and the social environment are considered. In particular, individuals strongly respond to religious norms concerning fertility, with the highest fertility rate observed in the less frequent religious group (i.e. Orthodox Christians, <10%). It is unlikely that norm violation is cost free, however. Theoretical modelling reveals that norms are stabilized with punishment mechanisms, for instance through reputational effects in which norm violators are sanctioned in receiving less help during subsequent interactions [52]. It is also possible that the effect of religion results not only (or not at all) from norm enforcement but from social transmission of attitudes and knowledge at religious gatherings. It is thus difficult to disentangle whether the effect of religion reflects an individual payoff assessment (balancing the cost of deviating from the norm with the benefit of contraceptive uptake) or social contagion among individuals.

Contraceptive uptake does not result from imitation of prestigious ever-users within friendships networks. The study cannot exclude the possibility that a woman's decision is influenced by prestigious individuals outside of the friendships networks. But given the low prevalence of contraceptive uptake in the population, prestige-bias is more likely to account for the persistence of high fertility rather than the spread of modern contraception. In this population, fertility restriction is unlikely to be associated with the ability to achieve success since there are few opportunities for education and no non-agricultural employment. However, in populations characterized by higher economic development, if the reduction in the number of children allows women to pursue education and thereby compete for high achieving jobs, a positive link between prestige-bias and contraceptive uptake is expected [6]. Nevertheless, the link between education and status is likely to be weak in populations entering the demographic transition and generally characterized by poor economic development [53]. One might thus suggest that if prestige-bias is important for reproductive decisions, it will slow down rather than speed up the early spread of low fertility norms.

Although we didn't find evidence of social transmission through friendships or spatial networks regarding the adoption of modern contraception, it is possible that women copy, learn or receive social support from other actors. First, women with many weak ties (e.g. ties with socially distant individuals) are more likely to adopt contraception. Weak ties bring knowledge not available through friends, and are usually found to be critical for the spread of ideas [46]. It is important to note, however, that the simple exposition to information does not appear to be sufficient for a woman to adopt modern contraception. Indeed, the results show that central women (i.e. those having more connections) are not more likely to adopt contraception, nor are women spatially close to ever-users. Second, it is likely that a woman's fertility decision is not independent of her husband's attitude. A study in Cambodia shows that women who believe that their husbands have a positive attitude towards modern contraception are >3 times more likely to use it [42]. Studies conducted both among the Mpimbwe of Tanzania [24] and in Gambia [54] revealed that women are more likely to effectively limit their family size using contraception if they don't have a constant spouse. Third, it has been suggested that women decisions regarding fertility practices could be influenced by their kin [55]. Among the Mpimbwe where mortality and fertility rates are high, women with a large number of siblings are more likely to be ever-users of birth control methods [24]. In the Gambia, however, little evidence was found that kin directly influence contraceptive uptake, either by their presence/absence or as models for social learning. Rather, contraceptive decisions in women were more directly related to socio-demographic variables such as age-specific parity and wealth rather than the presence or contraceptive behaviour of the extended family [54]. Overall, a woman's decision to adopt modern contraception will depend of potentially numerous sources of social transmission. Yet, whatever the source, one must identify the extent to which transmission refers to the behavioural imitation of others and/or information seeking. From an evolutionary perspective, these two mechanisms can reflect different selective pressures (i.e. cultural group selection and optimization of parental investment per child, respectively). Understanding which mechanism is at play at which stage of the transition will help to understand the underlying causes for the spread of low fertility practices.

To conclude, the initial slow uptake of low fertility norms is likely to be associated with socio-ecological conditions in which the use of modern contraceptives is not associated with reproductive advantages in the short term i.e. high mortality rate and low sibling competition. Only when conditions are met that favour contraception (e.g. competition between offspring for parental resources, high incentive to pursue education), social interactions are likely to be used to obtain information on the cost and benefits associated with the innovation. There is some evidence that the relevance of social learning in family planning networks varies with the level of development, and is more likely to be an important mechanism in an advanced market economy [14]. This is consistent with the view that market economies instil higher demand for parental investment in terms of education. It is also likely that the relevance of social influence vs. social learning varies with the prevalence of the cultural trait in the population, and if pure social influence through networks does not appear to explain the initial diffusion of low fertility norms, it is possible that this mechanism plays a more important role in the later maintenance of the trait. Our understanding of the processes and motivations underlying shifts to modern low fertility will only be advanced by recognizing the multiple stages of the demographic transition and by testing competing theoretical models simultaneously.

## Supporting Information

Table S1 **Characteristics of the villages in 2008.**
(DOC)

Table S2 **Best models for contraceptive uptake (individual factors only)**
(DOC)

Table S3 **Averaged estimates, standard errors and 95% confidence intervals based on models comparison (set 1, see methods section).** Models in set 1 only consider individual socio-economic and demographic characteristics. For each model, a logistic discrete hazard model with no intercept and time period as a main effect has been ran on a person to period dataset. Nomenclature: P: time period; Parity: number of living children. NA: Not applicable.
(DOC)

Table S4 **Best models for contraceptive uptake (analysis restricted to the last year of data)**
(DOC)
