# Correction: Social Transmission and the Spread of Modern Contraception in Rural Ethiopia

**DOI:** 10.1371/annotation/d4ace6be-2089-47e8-86b2-42d04189324a

**Published:** 2011-08-09

**Authors:** Alexandra Alvergne, Mhairi A. Gibson, Eshetu Gurmu, Ruth Mace

The order of the authors is incorrect in the byline. The correct author order is: Alexandra Alvergne, Eshetu Gurmu, Mhairi A. Gibson, Ruth Mace

The corrected citation is: Alvergne A, Gurmu E, Gibson MA, Mace R (2011) Social Transmission and the Spread of Modern Contraception in Rural Ethiopia. PLoS ONE 6(7): e22515. doi:10.1371/journal.pone.0022515

