# Supplementary material for: Social Transmission and the Spread of Modern Contraception in Rural Ethiopia
Source: PLoS One. 2011 Jul 22;6(7):e22515. doi: 10.1371/journal.pone.0022515 (PMC3142194; doi:10.1371/journal.pone.0022515)
Supplement: Table S1 — Characteristics of the villages in 2008. (DOC) [file pone.0022515.s001.doc]

**Supporting Information**

**Social transmission and the spread of modern contraception in rural Ethiopia**

**Alexandra Alvergne, Mhairi Gibson, Eshetu Gurmu and Ruth Mace**

**Table S1. Characteristics of the villages in 2008.**

|  | **Village of residence**  **A B C D** | | | | |
| --- | --- | --- | --- | --- | --- |
| Number of women of reproductive age | | **199** | **262** | **218** | **257** |
| Age (mean ± s.d.) | | 30.7 (8.3) | 32.2(8.8) | 31.0(9.2) | 32.7(8.8) |
| Husband’s cattle (mean ± s.d.) | | 2.2 (5.5) | 1.6 (1.3) | 2.0(1.6) | 1.3(1.0) |
| Agricultural productivity in $ (mean ± s.d.) | | 287.5 (193.4) | 269.7(203.6) | 266.8(147.7) | 155.9(102.4) |
| Nb. children produced (mean ± s.d.) | | 4.6 (2.8) | 4.6(2.7) | 5.1(2.8) | 5.0(2.9) |
| Nb. living children (mean ± s.d.) | | 3.6 (2.2) | 3.7(2.2) | 4.1(2.3) | 3.8(2.3) |
| Orthodox Christians (%) | | 0 | 38.2 | 0 | 0 |
| Muslims (%) | | 100 | 61.8 | 100 | 100 |
| Born in the same village (%) | | 18.6 | 25.3 | 19.6 | 16.5 |
| Have attended formal school (%) | | 23.6 | 31.7 | 14.2 | 20.6 |
| Have heard about contraception (%) | | 92.5 | 94.3 | 100 | 97.3 |
| Ever used contraceptives in 2008 (%) | | 22.3 | 15.4 | 22.5 | 22.8 |
| Currently use contraceptives (%) | | 6.7 | 6.5 | 7.8 | 14.0 |
