# Supplementary material for: Social Transmission and the Spread of Modern Contraception in Rural Ethiopia
Source: PLoS One. 2011 Jul 22;6(7):e22515. doi: 10.1371/journal.pone.0022515 (PMC3142194; doi:10.1371/journal.pone.0022515)
Supplement: Table S2 — Best models for contraceptive uptake (individual factors only) (DOC) [file pone.0022515.s002.doc]

**Supporting Information**

**Social transmission and the spread of modern contraception in rural Ethiopia**

**Alexandra Alvergne, Mhairi Gibson, Eshetu Gurmu and Ruth Mace**

**Table S2. Best models for contraceptive uptake (individual factors only)**

| **Models** | **K** | **LogLik** | **dAIC** | **ω_i_** |
| --- | --- | --- | --- | --- |
| Age+Age²+Cohort+Parity+Edu+MS | 21 | -808.20 | 0.00 | 0.49 |
| Age+Age²+Cohort+Parity+Edu+MS+ Nb_ DO | 22 | -807.69 | 0.98 | 0.30 |
| Age+Age²+Cohort+Parity+Edu+MS+Wealth | 23 | -807.45 | 2.53 | 0.14 |
| Age+Age²+Cohort+Parity+Edu+MS+Wealth+Nb_DO | 24 | -807.03 | 3.65 | 0.08 |

K: number of parameters; LogLik: Loglikelihood; dAIC : deviation from the best model’s AIC; ωi : Akaike weights; Parity: Number of living children; Edu: Level of Education; MS: Marital status; Nb_DO: Number of deceased offspring; Wealth: agricultural production + husband’s cattle.
