# Supplementary material for: Social Transmission and the Spread of Modern Contraception in Rural Ethiopia
Source: PLoS One. 2011 Jul 22;6(7):e22515. doi: 10.1371/journal.pone.0022515 (PMC3142194; doi:10.1371/journal.pone.0022515)
Supplement: Table S3 — Averaged estimates, standard errors and 95% confidence intervals based on models comparison (set 1, see methods section). Models in set 1 only consider individual socio-economic and demographic characteristics. For each model, a logistic discrete hazard model with no intercept and time period as a main effect has been ran on a person to period dataset. Nomenclature: P: time period; Parity: number of living children. NA: Not applicable. (DOC) [file pone.0022515.s003.doc]

**Supporting Information**

**Social transmission and the spread of modern contraception in rural Ethiopia**

**Alexandra Alvergne, Mhairi Gibson, Eshetu Gurmu and Ruth Mace**

**Table S3. Averaged estimates, standard errors and 95% confidence intervals based on models comparison (Set 1, see methods section).**

| **Variables** | | **Estimate ± S.E.** | **95% C.I.** |
| --- | --- | --- | --- |
| **P1** | | -10.06 ± 2.51 | [-14.97; -5.15] |
| **P2** | | -10.76 ± 2.58 | [-15.81; -5.71] |
| **P3** | | -10.59 ± 2.59 | [-15.67; -5.51] |
| **P4** | | -10.10 ± 2.61 | [-15.22; -4.98] |
| **P5** | | -9.26 ± 2.65 | [-14.45; -4.07] |
| **P6** | | -14.69 ± -4.10 | [-14.69; -4.10] |
| **P7** | | -9.15 ± 2.75 | [-14.53; -3.77] |
| **P8** | | -9.43 ± 2.79 | [-14.90; -3.95] |
| **P9** | | -9.22 ± 2.83 | [-14.78; -3.67] |
| **P10** | | -9.27 ± 2.87 | [-14.90; -3.64] |
| **P11** | | -9.25 ± 2.90 | [-14.94; -3.56] |
| **P12** | | -9.29 ± 2.92 | [-15.02; -3.57] |
| **P13** | | -9.08 ± 2.94 | [-14.84; -3.33] |
| **P14** | | -8.76 ± 2.96 | [-14.57; -2.95] |
| **Age** | | 0.14 ± 0.13 | [-0.13; 0.40] |
| **Age²** | | 0.00 ± 0.00 | [-0.01; 0.00] |
| **Parity** | | 0.36 ± 0.06 | [ 0.25; 0.48] |
| **Nb. deceased offspring** | | 0.08 ± 0.08 | [-0.08; 0.25] |
| **Prop. sons alive** | | 0.43 ± 0.23 | [-0.01; 0.88] |
| **Agricultural production** | | 0.00 ± 0.00 | [ 0.00; 0.00] |
| **Husband’s cattle** | | -0.06 ± 0.06 | [-0.19; 0.08] |
| **Educated** | | **----** | **----** |
| **Not Educated** | | -0.77 ± 0.17 | [-1.11; -0.43] |
| **Monogamous** | | **----** | **----** |
| **Unmarried** | | -1.35 ± 0.75 | [-2.82; -0.12] |
| **Polygynous** | | 0.20 ± 0.19 | [-0.16; 0.57] |
| **Cohort** | 1.03 ± 0.22 | | [ 0.60; 1.47] |

Models in set 1 only consider individual socio-economic and demographic characteristics. For each model, a logistic discrete hazard model with no intercept and time period as a main effect has been ran on a person to period dataset. Nomenclature: P: time period; Parity: number of living children. NA: Not applicable.
