# Supplementary material for: Social Transmission and the Spread of Modern Contraception in Rural Ethiopia
Source: PLoS One. 2011 Jul 22;6(7):e22515. doi: 10.1371/journal.pone.0022515 (PMC3142194; doi:10.1371/journal.pone.0022515)
Supplement: Table S4 — Best models for contraceptive uptake (analysis restricted to the last year of data) (DOC) [file pone.0022515.s004.doc]

**Supporting Information**

**Social transmission and the spread of modern contraception in rural Ethiopia**

**Alexandra Alvergne, Mhairi Gibson, Eshetu Gurmu and Ruth Mace**

**Table S4. Best models for contraceptive uptake (analysis restricted to the last year of data)**

| **Models** | **K** | **LogLik** | **dAIC** | **ω_i_** |
| --- | --- | --- | --- | --- |
| IF | 9 | -43.92 | 0.00 | 0.25 |
| IF + SI (Biased prop.innovators (popularity)) | 11 | -43.43 | 0.80 | 0.17 |
| IF + SI (Min.distance) | 10 | -43.88 | 1.92 | 0.09 |
| IF + SE | 14 | -39.93 | 2.03 | 0.09 |
| IF + SI (Centrality) | 11 | -43.05 | 2.27 | 0.08 |
| IF + SE + SI (Biased prop.innovators (popularity)) | 16 | -38.49 | 3.15 | 0.05 |
| IF + SE + SI (Min. distance) | 15 | -39.83 | 3.82 | 0.04 |
| IF + SI (Biased prop.innovators(education)) | 11 | -43.86 | 3.88 | 0.04 |
| IF + SI (Unbiased prop.innovators) | 11 | -43.86 | 3.88 | 0.04 |
| IF + SI (Biased prop.innovators(wealth)) | 11 | -43.87 | 3.90 | 0.03 |
| IF x SI (Min. distance) | 11 | -43.87 | 3.90 | 0.03 |
| IF + SE + SI (Centrality) | 16 | -39.19 | 4.54 | 0.03 |
| IF + SE + SI (Biased prop.innovators(education)) | 16 | -39.77 | 5.69 | 0.01 |
| IF + SI + SE (Unbiased prop.innovators) | 16 | -39.77 | 5.69 | 0.01 |

K: number of parameters; LogLik: Loglikelihood; dAIC : deviation from the best model’s AIC; ωi : Akaike weights; “x” indicates an interaction term.
